# Investigation of an Acute Gastrointestinal Illness Outbreak Linked to Drinking Water in a Higher Educational Institute in East Sikkim, India

**DOI:** 10.7759/cureus.64050

**Published:** 2024-07-07

**Authors:** Karma G Dolma, Madhuchhanda Das, Shanmuga S Saravanabhavan, Rachana Khati, Goutam Chowdhury, Jayden L Bhutia, Vizovonuo Visi, Rakesh K Saroj, Thandavarayan Ramamurthy

**Affiliations:** 1 Microbiology, Sikkim Manipal Institute of Medical Sciences, Sikkim Manipal University, Gangtok, IND; 2 Epidemiology and Communicable Diseases, Indian Council of Medical Research, New Delhi, IND; 3 Biotechnology, Aarupadai Veedu Institute of Technology Chennai Campus, Vinayaka Mission's Research Foundation, Chennai, IND; 4 Bacteriology, National Institute of Cholera and Enteric Diseases, Kolkata, IND; 5 Community Medicine, Sikkim Manipal Institute of Medical Sciences, Sikkim Manipal University, Gangtok, IND; 6 Community Medicine, North Eastern Indira Gandhi Regional Institute of Health and Medical Sciences, Shillong, IND; 7 Computational and Integrative Sciences, Jawaharlal Nehru University, New Delhi, IND

**Keywords:** polymicrobial infection, waterborne illness, outbreak, enteric pathogens, acute gastroenteritis

## Abstract

Introduction

An acute gastrointestinal illness outbreak was reported in a higher educational institution among students and faculties in East Sikkim, India, from January to February 2023. The investigation was conducted to identify the source of the infection and causative pathogens and prevent the spread of the outbreak.

Methods

We defined a case as three or more loose stools in 24 hours, abdominal pain, or vomiting with the onset of symptoms between January 16 and February 16, 2023. Active surveillance was conducted by reviewing the affected individuals at the campus and patient registers at the dispensary, where cases were treated. Stool samples, rectal swabs, water samples, and suspected food samples were collected for microbiological testing using conventional culture, multiplex polymerase chain reaction (PCR), and kit-based real-time PCR methods.

Results

Out of 1,850 residents, 106 (5.7%) were affected by gastrointestinal symptoms like diarrhea, vomiting, etc. The attack rate for females was 23 (1.24%) and for males was 83 (4.49%). The most affected individual median age was 21 years (range: 2-51 years). From the laboratory investigations, most of the cases demonstrated polymicrobial etiologies. Gastroenteritis pathogens like *Campylobacter*, astrovirus, androtavirusdiarrheagenic *Escherichia coli* (DEC) (EAEC, EIEC, ETEC, EPEC, and EAEC), *Shigella*,etc., were detected in the suspected samples. The environmental investigation indicated the presence of rusted and leaky water pipes and sewage pipelines, along with ineffective chlorination of the water plant.

Conclusions

Based on epidemiological and laboratory investigations, it is conjectured that sewage and fecal contamination of drinking water and poor maintenance of the water distribution system most likely caused the outbreak described in this study. Basic treatment modalities, adequate chlorination, and periodic inspection of the water system were suggested, which controlled the outbreak to a greater extent.

## Introduction

A foodborne disease outbreak (FBDO) is a public health emergency. A foodborne illness outbreak is defined as an incident where two or more people experience a similar illness resulting from the ingestion of a common food [1]. A quick investigation can reduce the mortality and morbidity caused by an outbreak. Every year, nearly one in 10 people worldwide falls ill after consuming contaminated food, resulting in more than 420,000 deaths and the loss of 33 million disability-adjusted life years [2]. In the WHO Initiative to Estimate the Global Burden of Food-borne Diseases, diarrheal agents were listed as the foremost source of 600 million foodborne infections and 420,000 deaths in 2010. Among them, the top-listed pathogens included *Salmonella, *hepatitis A virus, *Taenia solium*, etc. [3]. In India, the burden is particularly high, with more than 13.9 million cases reported in 2016 and 709 acute diarrheal disease (ADD) outbreaks reported, accounting for more than 25% of all outbreaks [4]. This is despite reports of high rates of foodborne infections, mostly in urban areas. For most FBDOs in India, the etiology is seldom identified due to the lack of timely laboratory diagnosis [5]. About 20% of all outbreaks reported are related to foodborne. Further information on the FBDO studies is exceptionally limited [6,7]. Even among the reported outbreaks, complete epidemiological and laboratory investigations are rare, and the sources of the outbreak are infrequently identified. Therefore, to identify the foodborne pathogens circulating in the northeast region and to investigate outbreaks systematically to generate evidence for appropriate public health action and policies, the Indian Council of Medical Research (ICMR) initiated a task force (TF) study on “Food borne disease pathogen surveillance in North-Eastern India” known as “ICMR FoodNet” (www.icmrfoodnet.in). Sikkim, one of the smallest states on the Indian continent, shares three distinct international borders. In this paper, we have reported a suspected foodborne outbreak on a higher educational campus among students and faculty from East Sikkim, India, between January 16 and February 16, 2023. To the best of our information, this is the first reported case of an acute gastroenteritis (AGE) outbreak caused by polymicrobial pathogens in East Sikkim.

## Materials and methods

Outbreak setting

On January 28, 2023, an increased number of ADD cases were reported from a higher educational campus in East Sikkim. The Department of Microbiology at Sikkim Manipal Institute of Medical Sciences (SMIMS) in Gangtok is one of the centers of the ICMR FoodNet TF project and has conducted this outbreak investigation along with the Department of Community Medicine, SMIMS to find out the source and causative pathogens of the outbreak and suggest recommendations for the control of the outbreak.

Case finding

We defined a case as three or more loose stools in 24 hours, abdominal pain, or vomiting with onset between January 16, 2023, and February 16, 2023, on the educational campus. We conducted passive surveillance by reviewing the medical register of the sick students and staff (including their families) at the campus dispensary where cases were treated. We carried out active case finding by conducting a comprehensive case search in hostels and staff quarters of the campus.

Data collection

A descriptive study was conducted by the foodborne investigation team by including all the students, faculties, staff, and their family members living on campus from January 16, 2023, and February 16, 2023. We excluded all cases suffering from abdominal pain, vomiting, or loose stools before the mentioned outbreak period. All the cases were interviewed using a pretested, semi-structured questionnaire. Details regarding sociodemographic, food, and water items consumed with their respective sources, symptoms, course of illness, and treatment sought were included in the questionnaire. The sociodemographic details include food exposure, time of food consumption, type of illness, date and time of onset of illness, clinical profile, and outcome. Every person present on campus during the outbreak period was enrolled in the study. A line list was prepared, and an epidemic curve was plotted by date and time of onset of AGE along with the calculation of age, gender, and food-specific attack rates. The hospitalization rate was also calculated. The clinical profiles of all AGE cases were recorded.

Laboratory investigations

We collected stool samples and rectal swabs from the symptomatic cases to isolate and identify the enteric pathogens by culture following the standard methods [8]. Suspected food samples, environmental samples, and swabs from food handlers in the hostel mess and canteen were collected. Furthermore, water samples collected from the various water tanks on campus were also tested. At least two swabs were collected from each individual. One was processed in the Department of Microbiology, SMIMS, by multiplex polymerase chain reaction (PCR). One rectal swab in phosphate buffered saline was transferred to the National Institute of Cholera and Enteric Diseases (NICED), Kolkata. In addition to enteric bacteria, enteric viruses (rotaviruses and adenoviruses) were also screened using commercial kits (TECHLAB, Inc., Radford, Virginia, United States) and (Vitassay Healthcare, S.L.U., Huesca, Spain). *Shigella* spp. isolates were identified using standard methods. Additionally, biochemically identified *Shigella* isolates were confirmed serologically by slide agglutination using commercially purchased antisera (Denka Seiken Co. Ltd., Tokyo, Japan). The antimicrobial susceptibility test was performed on Mueller-Hinton plates using the agar diffusion technique using commercial antibiotic discs (BD-BBL, United States). Based on the Clinical and Laboratory Standard Institute guidelines [9], the inhibition zone diameters recorded were resistant to an antimicrobial agent (CLSI, 2020). For quality control, strains like Escherichia coli (ATCC 25922) were used. All the E. coli confirmed by biochemical testing were screened for the presence of ETEC, EPEC, EIEC, and EAEC in the multiplex PCR assay following the protocol. In addition, the rectal swab was tested using the real-time PCR-based GastroFinder kit (Pathfinder, the Netherlands). The GastroFinder 2SMART, which is based on a multiplex PCR assay (Roche, Switzerland), helps in the concurrent identification and diversity of 18 gastrointestinal pathogens in less than three hours. The assay was performed by following the kit protocol. This test was carried out at NICED, Kolkata.

Environmental investigation

**Figure 1:**
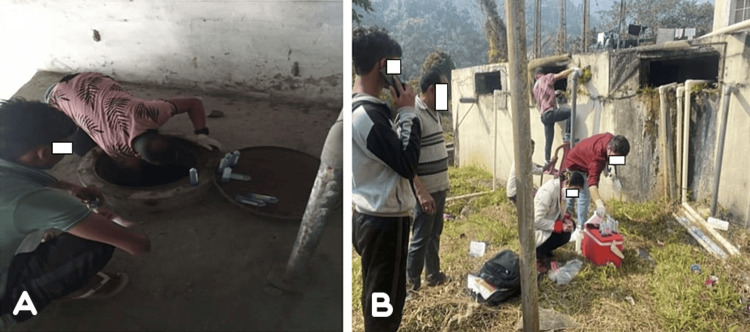
Water sample collection from (A) sump 3 behind hostel 3 and (B) sump 1 and 2 behind hostel 2 on the campus

Epidemiologists from the Department of Community Medicine and trained health workers in the ICMR FoodNet Project collected all sociodemographic information using a standardized, formulated AGE case search format. The mess’s kitchen, storage facility, and dining areas of the hostel were checked for cleanliness and sanitation. All aspects of the mess were inspected, from washing, cutting, storing, cooking, serving, and cleaning. All the food handlers were clinically examined to check for and identify any infections. The convenience and refuse disposal sites were also inspected. The water pipelines to the campus were also inspected for any leakage or cross-contamination with sewage lines. Four borewells (2, 3, 5, and 6) and three sumps (1, 2, and 3) were identified and inspected as the key sources of water supply for the residents of the campus (Figure 1). Four borewells and two sumps were located behind hostels 1 and 2 and the mess building. One sump was located behind the hostel 3 and 4 buildings, as shown in the spot map.

Data analysis

The food-specific attack rates were calculated for each food item. Univariate logistic regression analysis was carried out for food items with a P < 0.2 on univariate analysis using Epi Info version 7.2.0.1 (Centers for Disease Control, Atlanta, Georgia, United States). The attack rate was calculated as the total number of new cases divided by the total population. The food-specific attack rate was calculated as the total number of persons who ate a specified food and became ill divided by the total number of persons who ate that food [10].

Ethical considerations

The study was approved by the Ethics Committee of Sikkim Manipal University, Gangtok, India (approval number: SMIMS/IEC/2019-116) as a part of the TF study, in which outbreak investigation is one of the objectives. Informed written consent was obtained from each participant before enrolling the cases for data collection and the stool/rectal sample collection, where applicable. The investigation was aimed at achieving public good (beneficence) and collective welfare (solidarity); no harm was done to any individual (nonmaleficence); it was fair, honest, and transparent (accountability and transparency); and participants’ data were de-identified prior to analysis (confidentiality).

## Results

Descriptive epidemiology

**Figure 2:**
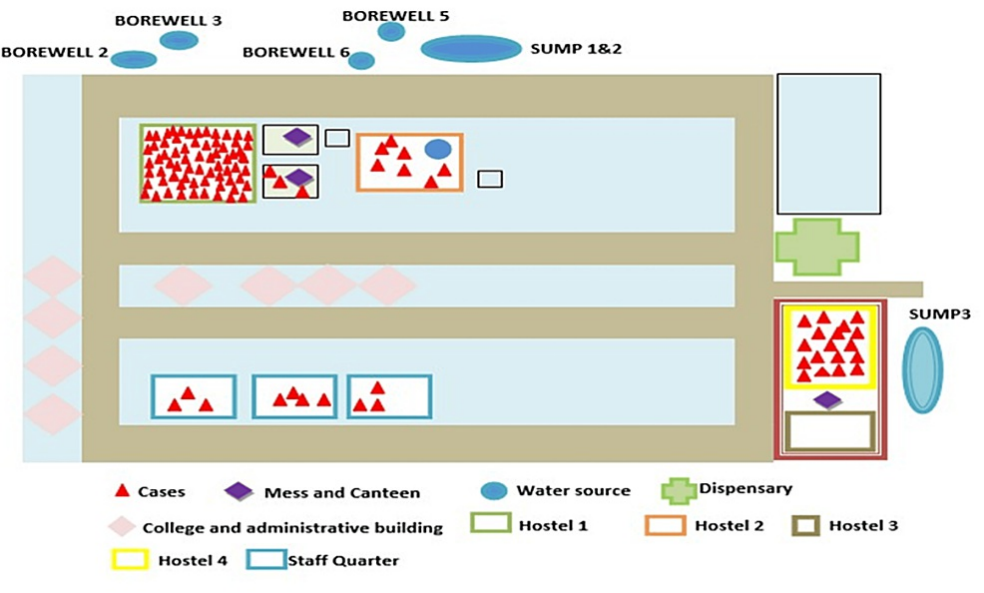
Spot map of diarrheal cases (red triangle) on the affected campus in East Sikkim

Overall, 106 of 1,850 (5.73%) infected and symptomatic cases from the affected area in East Sikkim were enrolled as participants and interviewed. Informed written consent was obtained from the cases before the initiation of the questionnaire. The questionnaire started on January 28 and was completed on February 16, 2023. Of the 106 participants, 91 (85.8%) were students, nine (8.5%) were staff, three (2.8%) were staff family members, and three (2.8%) were food handlers at the campus mess. Of the 106 affected residents, 23 (21.7%) were female, and 83 (78.3%) were male. The first affected individual showed the symptoms on January 16 at 8:00 a.m., the second case on January 18 at 10:00 p.m., and four more cases on January 21 at 7:00 a.m. to 11:00 a.m. The number of cases started increasing every day and peaked between January 22 and 30, 2023 (n = 83) between 6.00 and 8.00 a.m. Up to 15 people fell ill (median 1) per day during this period, and after this period, the outbreak ceased. The epidemic curve exhibited a continuous rise with a persisting upward slope followed by a steady descending slope, indicating a persevering common source (Figure 2).

The median age of the most affected individuals was 21 years (range: 2-51) (Table 1). The most predominant symptom was diarrhea, which was reported by all the affected individuals, of which 92 (86.8%) cases had moderate diarrhea and 14 (13.2%) suffered from severe diarrhea. A total of 20 (18.87%) people had diarrhea, vomiting, fever, abdominal cramps, and nausea. About 96 cases (90.56%) had only diarrhea and vomiting. Among the 23 affected females, 20 (87%) were suffering from moderate diarrhea, and three (13%) had severe diarrhea. Among males, 72 (86.7%) had moderate diarrhea, and 11 (13.3%) suffered from severe diarrhea. The attack rates were high among males: 83/106 (4.49%) compared to females: 23/106 (1.24%). The average age group of 21 years had the highest attack rate of 4.32% (80/106) (Table 1).

**Table 1:** Age and gender attack rate of diarrhea cases during the outbreak

Age group	Case (n = 106)	Total population (n = 1,850) attack rate (%)	Female (n = 23)	Attack rate	Male (n = 83)	Attack rate
2-11	2	0.11	0	0	2	0.11
12-21	80	4.32	14	0.76	66	3.57
22-31	15	0.81	3	0.16	12	0.65
32-41	4	0.22	3	0.16	1	0.05
42-51	5	0.27	3	0.16	2	0.11
Grand total	106	1.24	23	1.24	83	4.49

In the distribution of affected individuals, the largest number of cases was from hostel 1 (68 cases, 64.15%), followed by hostel 4 (18 cases, 16.9%), staff quarter (10 cases, 9.43%), hostel 2 (seven cases, 6.63%), and campus mess (three cases, 2.83%), as shown in Figure 2. Clustering was mainly observed in hostel 1, and other cases were observed to be scattered around the campus in different areas like hostel 2, hostel 4, and the staff quarter.

Each specific food item was analyzed for the specific food attack rates consumed by the cases (Table 2). Considering the time of consumption of water as the exposure time (12:00 p.m. on January 16, 2023), we estimated the median incubation period of the infection as eight hours (range: 0-45 hours). Only 65 (61.3%) individuals consumed rice, 59 (55.7%) consumed dal, 90 (84.9%) drank water, and 15 (14.2%) consumed roti (Table 2). The majority of the cases (86.8%) had moderate diarrhea, whereas 14 (13.2%) suffered from severe diarrhea. As shown in Table 2, severe diarrhea was found in those who consumed rice (10.8%), dal (lentils) (8.5%), sabji (mixed vegetables) (6.3%), and roti (flat bread) (6.7%). About 80 (88.9%) people who consumed water had moderate diarrhea, and 10 (11.1%) had severe diarrhea.

**Table 2:** Food-specific attack rate

Variables, n (%)		Moderate diarrhea (%)	Attack rate	Severe diarrhea (%)	Attack rate
Gender	Female, 23 (21.7)	20 (87)	1.08%	3 (13)	0.16%
	Male, 83 (78.3)	72 (86.7)	3.89%	11 (13.3)	0.59%
Total		92 (86.8)	4.97%	14 (13.2)	0.75%
Water	No, 16 (15.1)	15 (93.75)	-	1 (6.25)	-
	Yes, 90 (84.9)	80 (88.88)	4.32%	10 (11.11)	0.55%
Total		92 (86.8)	4.32%	14 (13.2)	0.55%
Rice	No, 41(38.7)	34 (82.9)	-	7 (17.1)	-
	Yes, 65 (61.3)	58 (89.2)	3.13%	7 (10.8)	0.378%
Total		92 (86.8)	3.13%	14 (13.2)	0.378%
Dal	No, 47 (44.3)	38 (80.9)	-	9 (19.1)	-
	Yes, 59 (55.7)	54 (91.5)	2.91%	5 (8.5)	0.27%
Total		92 (86.8)	2.91%	14 (13.2)	0.27%
Sabji	No, 74 (69.8%)	62 (83.8)	-	12 (16.2)	-
	Yes, 32 (30.1%)	30 (93.8)	1.62%	2 (6.3)	0.10%
Total		92 (86.8)	1.62%	14 (13.2)	0.10%
Roti	No, 91 (85.8)	78 (85.7)	-	13 (14.3)	-
	Yes, 15 (14.2)	14 (93.3)	0.75%	1 (6.7)	0.054%
Total		92 (86.8)	0.75%	14 (13.2)	0.054%

Environmental investigation

The environmental survey of the campus mess was also carried out, and it was observed that hygiene was well maintained in the hostel mess area, where cleaning of the area was done after every meal. The kitchen staff also wore head covers or gloves at the time of the visit. The prepared food was all covered, and raw materials were kept in the store near the kitchen. The poultry was sourced locally and was not kept for more than 24 hours. All the expired products were kept on separate shelves, and dry products like rice, flour, etc., were kept on an elevated platform. The fumigation pest control record was also checked, and it was observed that pest control was done at regular intervals (twice a month).

Drinking water on campus, supplied from four borewells and three sumps, was investigated in terms of location and surroundings. The untreated water from the sumps (1, 2, and 3) was supplied to the reverse osmosis (RO) treatment plant, located above hostel 1, 3, and 4 buildings. This RO-treated water was the main water source, which supplied water to all the buildings on the campus. Three (16.6%) RO filter systems were also installed in the mess. It was observed that ninety residents (84.9%) affected individuals used this particular RO water for drinking purposes. On inspecting the drinking water lines to the hostel and around the hostels, all the pipes were intact; however, they appeared very rusted and dirty-looking, with prominent leaks observed in a few places. Further, the sewage pipes could not be accessed properly as they were underground. The only connecting pipes coming out of the toilets could be seen; however, these pipes also went underground, connecting to the main sewage line. We further observed that some sewage pipelines were right near sump 1 and 2, with some foul smells near the area.

Laboratory results

Among the 106 cases, only 22 rectal swabs, 11 stool samples, 18 water samples, 29 food samples, and seven environmental samples were collected for analysis.

GastroFinder Method for Rectal Samples

Out of 22 rectal swabs, 17 were positive (10 polymicrobial infections and seven monomicrobial infections), and five swabs were negative. The pathogen with the highest isolation was *Campylobacter *spp. (12), followed by astrovirus (eight), EIEC/*Shigella *(six), ETEC (three), and one adenovirus.

Multiplex PCR for Rectal and Stool Samples

Out of 22 rectal swabs, we were able to identify EAEC (15) and EPEC (6). From 11 stool samples, five were positive for EAEC, and the remaining six were negative for diarrheagenic strains of *E. coli*. One sample was positive for rotavirus using the Vitassay kit.

Water Samples

Of the 18 water samples collected, 12 were positive for *E. coli *by conventional culture and biochemical analysis. Out of this *E. coli*, 11 were positive for EAEC, three for EPEC, two for EHEC, and one for ETEC. Three water samples were positive for both EAEC and EPEC, two water samples were positive for both EAEC and EHEC, and one water sample was positive for EAEC and ETEC.

Food and environmental samples

**Figure 3:**
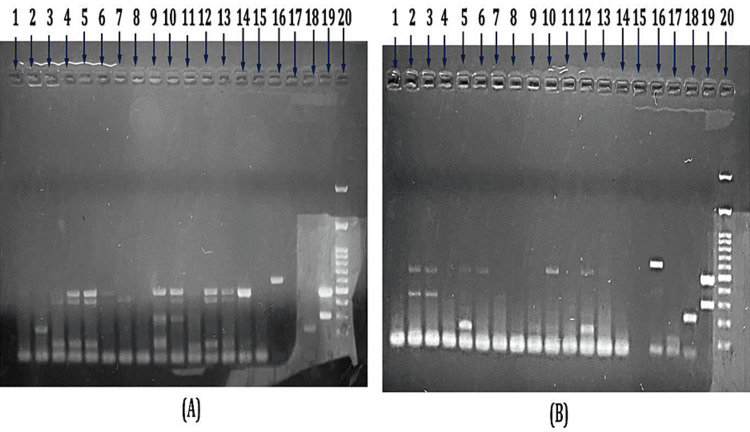
Multiplex PCR of the reference strain and tested samples (A and B) Lane 1-14: tested sample; Lane 15: negative control; Lane 16: reference strain EHEC (stx1 amplicon size 348 bp and stx2 amplicon size 584 bp); Lane 17: reference strain EAEC (aafII amplicon size 378 bp and astA ICRV 181 amplicon size 102 bp); Lane 18: reference strain ETEC (elt amplicon size 218 bp and estII amplicon size 129 bp); Lane 19: reference strain EPEC (eaeA amplicon size 482 bp and BFP amplicon size 300 bp); Lane 20: DNA molecular size marker (1,000 bp ladder) PCR, polymerase chain reaction

Twenty-nine cooked food items were collected from the mess and canteen for microbial analysis. Seven environmental swabs of the chopping board, working table, roti roller, and taps were collected. In the environmental samples, five samples were positive for EAEC, and two samples showed a mixed infection of EAEC and EHEC (Figure 3).

Food handlers

Thirteen food handlers were contacted, and nail swabs were collected. Among 13 samples, only two (15.4%) *E. coli* were detected, but it was negative for the DEC in the multiplex PCR (Table 3).

**Table 3:** Laboratory findings of the various specimens collected from the site PCR, polymerase chain reaction; RO, reverse osmosis

Sample type	Method	Positive	Organism
Rectal swab (22)	GastroFinder (Real time PCR)	12	*Campylobacter *spp.
		8	Astrovirus
		6	ipaH (EIEC/*Shigella*)
		3	elt/est (ETEC)
		1	Adenovirus
	Polymicrobial infection detection by GastroFinder	6	*Campylobacter *spp. and astrovirus
		4	ipaH (EIEC/*Shigella*) and *Campylobacter *spp.
		3	ipaH (EIEC/*Shigella*) and astrovirus
		1	elt/est (ETEC) and *Campylobacter* spp.
		1	*Campylobacter *spp., ipaH (EIEC/*Shigella*), and astrovirus
		1	*Campylobacter *spp., ipaH (EIEC/*Shigella*), adenovirus, and astrovirus
Rectal swab (22)	Conventional culture and biochemical identification	15	Escherichia coli
		3	*Klebsiella *spp.
		1	*Morganella* spp.
		1	Proteus mirabilis
		2	No growth
	Identification followed by multiplex PCR	15	EAEC: 15 EPEC: 06
Stool (11)	Conventional culture and biochemical identification	5	E. coli
		2	*Enterobacter *spp.
		2	*Citrobacter *spp.
		1	*Acinetobacte*r spp.
		1	Proteus vulgaris
	Identification followed by multiplex PCR	5	EAEC:05
	Vitassay kit	1	Rotavirus
Water samples (18) (4 = borewell, 3 = sump, 8 = tap water, and 3 = RO filtered water)	Membrane filtration method and culture and biochemical identification	12	E. coli
		2	*Klebsiella *spp.
		1	Proteus mirabilis
		2	*Stenotrophomonas *spp.
		1	No growth
	Identification followed by multiplex PCR	11	EAEC: 11
			EPEC:03
			ETEC:01
			EHEC:02
Environmental samples and food samples (36) (29 = cooked food items, 2 = roti roller swab, 3 = chopping board swab, 1 = table surface swab, and 1 = tap swab)	Conventional culture and biochemical identification	5	E. coli
		9	*Klebsiella *spp.
		1	*Staphylococcus *spp.
		5	*Acinetobacter *spp.
		6	No growth
		5	*Enterobacter *spp.
		2	*Pseudomonas* spp.
		2	Streptococcus
		1	Proteus vulgaris
	Identification followed by multiplex PCR	5	EAEC: 05 EHEC:01

Antibiotic susceptibility pattern of the DEC

**Figure 4:**
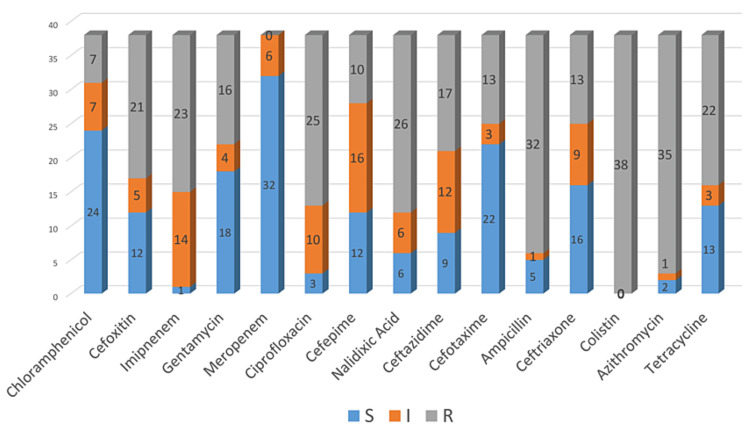
Overall antibiotic susceptibility of Escherichia coli (n = 38) isolated from patients, food samples, water samples, and environmental samples

The antibiotic susceptibility testing pattern of DEC showed the highest resistance (100%) to colistin, followed by ampicillin (83.3%), azithromycin (91.6%), ciprofloxacin and nalidixic acid (66.6% for both), imipenem (58.3%), tetracycline and cefoxitin (55.5% each), ceftazidime (47%), gentamicin (41.6%), cefotaxime and ceftriaxone (36% each), cefepime (27.7%), and chloramphenicol (19%). However, meropenem was found to be 100% sensitive (Figure 4).

Case management

Of the 106 cases, 92 (86.8%) consulted a physician in the dispensary, whereas 14 (13.2%) adopted self-medication. The cases were treated with oral fluids (74, 69.8%), antibiotics (71, 67%), antispasmodics, and antacids (37, 35%). The patients were treated with oral ofloxacin and ornidazole, lomofen, flagyl, and oral rehydration salt. The cases admitted were given intravenous levofloxacin, pantop, fevridol, and intravenous fluids. There were no deaths reported. Based on the information received from the students, some reported taking medicines as per the instructions from their parents. Overall, all patients were given first aid when they came to the dispensary.

## Discussion

An investigation was conducted into the outbreak of AGE that affected 5.7% of residents of a higher educational campus in East Sikkim. We outlined the specifics of an outbreak of AGE based on the medical indications, epidemiological characteristics, and laboratory outcomes. The outbreak occurred persistently for almost four weeks. The majority of the disease took place between January 21 and 29, 2023, over a period of 10 days. The study findings in this investigation suggest that the outbreak might have taken place due to the contamination of the water pipes with the sewage pipelines, resulting in the transmission of waterborne outbreaks in most of these cases.

Of the 106 cases identified in this study, 90 had consumed water from the RO water treatment plant supplied in the hostel area; around 16 cases had consumed food prepared from the same water. The standard precautionary measure to prevent diarrheal disease is the chlorination of water [11]. On campus, chlorination was done mainly in one of the RO water treatment plants; we can assume that the chlorination process was probably not done efficiently. Moreover, studies have shown that a single point of chlorination may not be effective enough to provide the basic chlorination level throughout the water distribution periphery [12]. Other methods of safe drinking water, like point-of-use chlorination and boiling water before consumption, should be practiced by all individuals [13,14].

In the laboratory findings, multiple pathogens, including *Campylobacter*, *Shigella *spp., EIEC, ETEC, EAEC, EPEC, ETEC, *Proteus mirabilis*, astrovirus, and rotavirus, were involved in the outbreak and were isolated from patients, water, environment, and food samples. Results of environmental and laboratory investigations indicated that the source of water was contaminated prominently with DEC (EAEC, EPEC, ETEC, and EHEC). Among the DEC pathotypes, it was also observed that EAEC was the most prominent type (rectal swab = 15, stool = 5, water sample = 11, and environmental samples = 5), followed by EPEC (rectal swab = 6 and water sample = 3) and EHEC (water sample = 2 and environmental samples = 1), confirmed by multiplex PCR. In certain low-income countries, some of the common etiological pathogens causing various ranges of diarrhea include rotavirus, *E. coli*, *Vibrio cholera*, etc. [15]. The occurrence of a polymicrobial outbreak is a rare event not reported often, especially from a place like Sikkim. The pathogenic agents involved in these kinds of outbreaks vary. The isolation and identification of various pathogenic microorganisms using various methods (conventional, rapid kits, and molecular) have reported polymicrobial etiologies. Generally, we see that investigations like this report only one or a dual pathogen as a source [16,17]. The outbreak may have resulted from fecal contamination of water supplied through leaky pipes, where all these pathogens have thrived together and caused the outbreak.

Based on the large number of cases being exposed to water and considering the fact that multiple pathogens were involved, water could possibly be associated with the source of infection. Many studies link that waterborne epidemics are associated with single or multiple pathogens indicating entry of sewage or feces into the drinking water supply system. Some outbreaks indicate contamination of groundwater occurs due to heavy rainfall and agricultural or industrial runoff [18]. Foodborne outbreaks caused by mixed pathogens have been reported from various places [19-21]. Here, we did find the drinking water pipes and the sewage pipes to be rusted and leaky in certain areas. Also, the location of the sewage pipelines was next to the sump area, where the drinking water was being supplied.

This investigation reemphasizes the importance of frequent water quality monitoring of the water supply, and proper chlorination measures should be in place. Additionally, a routine water quality monitoring system can identify water contamination and serve as an early warning source. Based on laboratory findings, the authorities of the educational campus were told to take prompt public health action as soon as possible. As a result, the entire water system on campus was immediately chlorinated, and regular quality checks of both water and food were conducted. Additional RO water filters with UV treatment were installed in the hostel or mess, and the quality of the water, as well as any leakages in the water and sewage lines, were regularly monitored and promptly repaired. Due to this action by the authorities, the incidence of the outbreak was soon controlled. The food handlers were also advised to take special care to follow good kitchen hygiene practices, particularly handwashing. There are certain limitations to our study. We could not enroll all the residents of the campus for their detailed information to conduct a case-control study as many residents refused, especially the students, citing various reasons like preparation for the upcoming exams, not being sick, and many having also left for home in order to avoid infection. Hence, we could not have a case-control study, which could have helped in the analysis significantly. Secondly, the incident of the outbreak was also informed at least a week late to the investigators. This, in some way, may have delayed investigations and prolonged identification and treatment in many cases.

## Conclusions

In this study, we report and confirm that the AGE outbreak in East Sikkim was due to leaky water and sewage pipelines and an additional factor of improper chlorination of the drinking water, leading to a polymicrobial outbreak of gastroenteritis on the campus. The investigators suggested basic treatment modalities should be provided en masse to all populations in the survey areas, like oral rehydration solutions, zinc tablets, and antibiotics. RO-filtered water and chlorinated water reservoirs should be cleaned and replaced at regular intervals. Additionally, aquaguards and UV filters for drinking water should also be installed in the affected area. Appropriate preventive measures in the form of extension activities like the incorporation of an active surveillance system with epidemiological and laboratory support can help to prevent mass-scale outbreaks in the near future.

## Appendices

**Table 4:** Percentage details of the variables

Variables	Number	Percentage
Gender		
Female	23	21.7
Male	83	78.3
Total	106	100.0
Rice		
No	41	38.7
Yes	65	61.3
Total	106	100.0
Dal		
No	47	44.3
Yes	59	55.7
Total	106	100.0
Water		
No	30	28.3
Yes	76	71.7
Total	106	100.0
Roti		
No	91	85.8
Yes	15	14.2
Total	106	100.0
Diarrhea details		
Moderate diarrhea	92	86.8
Severe diarrhea	14	13.2
Total	106	100.0

**Table 5:** Chi-square test analysis of diarrhea severity with other variables

Variables		Moderate diarrhea	Severe diarrhea	Total	Chi-square/Fisher exact test	Relative risk
Gender	Female	20 (87%)	3 (13%)	23 (100%)	0.642	1.00
	Male	72 (86.7%)	11 (13.3%)	83 (100%)		
Total		92 (86.8%)	14 (13.2%)	106 (100%)		
Rice	No	34 (82.9%)	7 (17.1%)	41 (100%)	0.259	0.929
	Yes	58 (89.2%)	7 (10.8%)	65 (100%)		
Total		92 (86.8%)	14 (13.2%)	106 (100%)		
Dal	No	38 (80.9%)	9 (19.1%)	47 (100%)	0.093	0.883
	Yes	54 (91.5%)	5 (8.5%)	59 (100%)		
Total		92 (86.8%)	14 (13.2%)	106(100%)		
Sabji	No	62 (83.8%)	12 (16.2%)	74 (100%)	0.139	0.894
	Yes	30 (93.8%)	2 (6.3%)	32 (100%)		
Total		92 86.8%)	14 (13.2%)	106 (100%)		
Water	No	26 (86.7%)	4 (13.3%)	30 (100%)	0.603	0.998
	Yes	66 (86.8%)	10 (13.2%)	76 (100%)		
Total		92 (86.8%)	14 (13.2%)	106 (100%)		
Roti	No	78 (85.7%)	13 (14.3%)	91 (100%)		
	Yes	14 (93.3%)	1 (6.7%)	15 (100%)		
Total		92 (86.8%)	14 (13.2%)	106 (100%)		

**Table 6:** Chi-square analysis of diarrhea with age

Diarrhea details		N	Mean	SEM	p-value
Age	Moderate diarrhea	92	22.04	0.682	0.641
	Severe diarrhea	14	21.14	2.083	

**Table 7:** OR analysis of variables

Variables	B	SE	p-value	OR	95% CI	
					Lower	Upper
Age	-0.013	0.051	0.803	0.987	0.893	1.092
Gender (1)	0.116	0.756	0.878	1.123	0.255	4.944
Rice (1)	-0.104	0.795	0.896	0.901	0.190	4.285
Dal (1)	0.873	0.823	0.289	2.395	0.477	12.022
Sabji (1)	0.721	0.845	0.394	2.056	0.392	10.779
Water (1)	-0.097	0.657	0.882	0.907	0.250	3.290
Roti (1)	0.684	1.122	0.542	1.981	0.220	17.865

**Table 8:** Relative risk analysis of the severity with other variables

Variables		Diarrhea details		Total	Relative risk
		Moderate diarrhea	Severe diarrhea		
Gender	Female	20	3	23	1.00
	Male	72	11	83	
Rice	No	34	7	41	0.892
	Yes	58	7	65	
Dal	No	38	9	47	0.883
	Yes	54	5	59	
Sabji	No	62	12	74	0.894
	Yes	30	2	32	
Water	No	26	4	30	0.998
	Yes	66	10	76	
Roti	No	78	13	91	0.918
	Yes	14	1	15	
